# Biochemical characterization of bioinspired nanosuspensions from *Swertia chirayita* extract and their therapeutic effects through nanotechnology approach

**DOI:** 10.1371/journal.pone.0293116

**Published:** 2024-02-08

**Authors:** Ayesha Raza, Tayyab Ali, Muhammad Naeem, Muhammad Asim, Fatma Hussain, Zhiye Li, Abdul Nasir

**Affiliations:** 1 Clinico-Molecular Biochemistry Laboratory, Department of Biochemistry, Faculty of Sciences, University of Agriculture, Faisalabad, Pakistan; 2 College of Life Science, Hebei Normal University, Shijiazhuang, China; 3 Department of Pharmacy, Second Affiliated Hospital of Zhengzhou University, Zhengzhou, Henan, China; 4 Medical Research Center, Second Affiliated Hospital of Zhengzhou University, Zhengzhou, Henan, China; University of Veterinary and Animal Sciences, PAKISTAN

## Abstract

*Swertia chirayita* is used as a traditional medicinal plant due to its pharmacological activities, including antioxidant, antidiabetic, antimicrobial, and cytotoxic. This study was aimed to evaluate the therapeutic efficacy of newly synthesized nanosuspensions from *Swertia chirayita* through nanotechnology for enhanced bioactivities. Biochemical characterization was carried out through spectroscopic analyses of HPLC and FTIR. Results revealed that extract contained higher TPCs (569.6 ± 7.8 mg GAE/100 g)) and TFCs (368.5 ± 9.39 mg CE/100 g) than *S. chirayita* nanosuspension, TPCs (500.6 ± 7.8 500.6 ± 7.8 mg GAE/100 g) and TFCs (229.5± 3.85 mg CE/100 g). Antioxidant activity was evaluated through DPPH scavenging assay, and nanosuspension exhibited a lower DPPH free radical scavenging potential (06 ±3.61) than extract (28.9± 3.85). Anti-dabetic potential was assessed throughα-amylase inhibition and anti-glycation assays. Extract showed higher (41.4%) antiglycation potential than 35.85% nanosuspension and 19.5% α-amylase inhibitory potential than 5% nanosuspension. Biofilm inhibition activity against *E. coli* was higher in nanosuspension (69.12%) than extract (62.08%). The extract showed high cytotoxicity potential (51.86%) than nanosuspension (33.63%). These nanosuspensions possessed enhanced bioactivities for therapeutic applications could be explored further for the development of new drugs.

## 1. Introduction

Several drugs and synthetic compounds have been used for the treatment of infectious diseases. However, excessive use of medications has several side effects, such as organ damage and cellular toxicities [1]. For example, cisplatin enhances the risk of nephron malfunctioning, liver damage, and gastric toxicity [2]. In contrast, benzofuran causes visual hallucinations and panic attacks [3]. Therefore, there is an urgent need to search for medicines to overcome these side effects. Therefore, medicinal plants and their extracts are excellent sources of bioactive compounds such as antioxidants, alkaloids, phenols, and flavonoids [4]. These bioactive compounds have been tested in clinical trials and thus possess therapeutic potential for the treatment of different diseases [5].

*Swertia chirayita* is used as a traditional medicinal plant and belongs to the Gentianaceae family. It is abundantly present in the temperate Himalayas region and used against different infectious diseases due to the presence of bioactive compounds [6]. The *S. chirayita* roots are used to treat leprosy, cholera, liver problems, and joint discomfort. The medicinal benefits of *S. chirayita* leaves have antiviral, analgesic, hypoglycemic, and antimalarial characteristics. *S. chiravita* also possesses anti-cancer, antimicrobial, and anti-allergic activities [7]. It also reduces blood sugar levels through its impact on insulin release [8].

Several studies have reported on the synthesis and formulations of nanosuspensions in many medicinal plants such as *Terminalia arjuna* [9], *Ginkgo biloba* [10], *Mentha arvensis L* [11], *Allium cepa* [12], and *Nigella sativa L*. [13]. These studies have shown promising results in increasing the bioavailability of natural bioactive compounds. However, there is little information available on the compositions of *S. chirayita* novel nanosuspension. As a result, more research is needed to investigate the possible function of higher bioavailability of natural bioactive chemicals in this plant’s nanosuspensions.

Nanotechnology has shown promising results in improving the extraction, purification, and bioavailability of natural products found in medicinal plants. Nanoparticles possess exceptional chemical, optical, and thermal properties and have become hopeful options for various biological applications [14]. One such approach that has shown improved absorption of medicines with low solubility is through the use of nanosuspension technology [15]. Nanosuspensions are used to produce nano-delivery systems that can increase the ability of hydrophobic medications to dissolve properly in an aqueous solution, thereby increasing their bioavailability [16].

The interest in using nanosuspensions for therapeutic applications has increased in drug delivery. With the help of nanosuspensions, particle sizes are reduced, increases drug delivery to target areas. It also improves stability after curing and enhances drug adhesion. Such nanosuspension characteristics have greatly increased the bioavailability and effectiveness of hydrophobic medicines [17]. Nanosuspensions are currently used for revolutionary cancer therapies and nano-drug delivery systems for fighting cancer and inflammatory diseases [13].

This study was aimed to evaluate the antioxidant, antidiabetic, antimicrobial, and cytotoxic activities of newly synthesized nanosuspensions from *Swertia chirayita* through nanotechnology. Biochemical characterization was carried out through spectroscopic analyses of HPLC and FTIR. These cheap, readily available methods could serve as potential sources for enhanced bioactivities of novel phytochemicals.

## 2. Material and methods

### 2.1 Chemical and reagents

Phenolic acids (benzoic acid, chlorogenic acid, salicylic acid, kaempherol, gallic acid, vanillic acid, and quercitin), Folin-Ciocalteu reagent, and 2,2-diphenyl-1-picrylhydrazyl (DPPH) were procured from Sigma-Aldrich Co. (St. Louis, United States). The source of bovine serum albumin was Alpha-Aesar Co. in the United States, whereas Merck Germany provided polyvinyl alcohol (PVA). Merck (Darmstadt, Germany) provided the solvents and chemicals for HPLC analysis. Analytical-grade materials were utilized throughout the research.

### 2.2 Plant material collection and extract preparation

*Swertia chirayita* was purchased from the registered market of Faisalabad and confirmed by the Department of Botany at the University of Agriculture in Faisalabad, Pakistan. After drying and grinding, the plant was crushed into powder, then stored in a clean, covered jar. The extraction of the powdered sample was performed at room temperature, and fatty substances were removed by the process of maceration with petroleum ether. The extraction was made by the Soxhlet apparatus., where the solvent was 95% ethanol [18]. The extract was kept at room temperature for evaporation, then taken in falcon tubes and stored in the freezer at -18°C.

### 2.3 Structural characterization

#### 2.3.1 High-performance liquid chromatography

*S. chirayita* whole plant extract that had been dried and hydrolyzed and used for HPLC analysis. In sample preparation, dried material of 0.5 grams was dissolved into 20 mL of 70% ethanol. After that, addition of 10 mL of 1M HCl was done, and the solution was then gently stirred. It was then placed in a water bath and refluxed for two hours at 90°C. The final mixture underwent HPLC analysis after refluxing. At a wavelength of 280 nm, measurements were taken after injecting a 20 μL sample. The corresponding retention times of different compounds were determined. The apparatus used for HPLC analysis was Flexar FX-20 with a C-18 column [19].

#### 2.3.2 FTIR spectroscopy

In order to understand the structural and functional groups present in the powdered *S. chirayita* plant, Fourier Transform Infrared Spectroscopy (FTIR) was applied. The sample was initially subjected to bireduction in a solution containing chloroauric acid and centrifugation was performed on the resultant solution for 15 minutes at a speed of 10,000 rpm. Then, 20 mL of deionized water was used to wash the collected pellet three times to eliminate any undesirable proteins or enzymes that were not attached to the particles. Agilent Cary 630 FTIR instrument was employed for the FTIR analysis, using a diffuse reflect-array mode and a 4 cm resolution. To achieve the desired signal/noise ratio, a total of 512 scans were completed [20].

### 2.4 Nanosuspension preparation

The nanoprecipitation technique was applied for the synthesis of nanosuspensions. One gram of extract was initially dissolved in 7.5 mL of a 3:1 solution of acetone and ethanol. By gradually infusing the solution into 10 mL of water, an emulsion was created, and the mixture was stirred at 1000 rpm constantly. The resultant emulsion was diluted in 20 mL of PVA solution (0.2% w/v in water) to avoid fusion and bubbling. The solution was stirred for 3 hours at room temperature at 500 rpm. This enabled the evaporation of the solvent and led to formation of nanosuspension. At -18°C, the nanosuspension was freeze-dried for further use [18].

### 2.5 Antioxidant profile

#### 2.5.1 Total phenolic contents (TPCs)

Phenolic contents were measured by using the Folin-Ciocalteu reagent. First, 125 μL test samples were mixed with 25 μL of 10% diluted Folin-Ciocalteu reagent. After that, 3mL Na_2_CO_3_ (1 percent w/v) was added to the solution and was incubated at 37°C for two hours. The absorbance was measured at 750 nm through a spectrophotometer and the results were calculated as mg GAE/g using the calibration curve of gallic acid [21].

#### 2.5.2 Total flavonoid content (TFCs)

By the AlCl_3_ colorimetric approach, contents of flavonoid were measured. In 96-well plates, a mixture of 9.5 μL of NaNO_2_ and 1.56 mL of distilled water was combined with the test samples (38 μL each). Then, 19 μL of 10% AlCl_3_ was poured to the solutions and incubated for almost 5 minutes. The optical density (OD) was then assessed using a twin-beam UV/Visible spectrophotometer at about 510 nm. Finally, using the calibration curve for catechin, the total contents of flavonoid in the nanosuspension and extract was determined [22].

#### 2.5.3 DPPH radical scavenging assay

When determining the antioxidant capacity, the 2,2-Diphenyl-1-picrylhydrazyl (DPPH) technique is a popular, quick, easy, and economical approach. It assesses a substance’s capacity to function as a free radical scavenger (FRS) or hydrogen donor using free radicals. This assay uses the DPPH testing process function for capturing the free radicals. For the antioxidant potential of nanosuspensions and extract, DPPH assay was performed. By abiding by standard regulations, 250 μL of DPPH solution was mixed with 2.5μL of extracts and nanosuspension solutions. Aluminum foil was used to cover resulting mixture and left for 35 minutes. Finally measured absorbance at 520 nm by spectrophotometer [13]. Percentage DPPH scavenging was calculated as:

$$\%\;\text{DPPH}\;\text{scavenging}=\left[A\left(\text{control}\right)–A\left(\text{sample}\right)/A\left(\text{control}\right)\right]\times 100$$

### 2.6 Biofilm formation inhibition assay

The following procedures were followed throughout the quantitative and qualitative biofilm inhibition experiments:

#### 2.6.1 Biofilm inhibition assay

A biofilm inhibition assay was used to assess antibacterial efficacy. 100 μL of *Staphylococcus aureus* and *Escherichia coli* with samples and nutrient broth were added to 96 wells plate, followed by aerobic incubation at 37°C overnight. Phosphate buffered saline (pH = 7.40) was used to rinse the plates three times and agitated to remove any adhering bacteria. Afterward, crystal violet stain 100 μL (50%) was applied precisely and any excess stain was eliminated by rinsing with tap water. The absorbance was subsequently measured using a plate reader (BioTek, USA) at 630 nm [11]. The positive control in experiment was ciprofloxacin, whereas the negative control was nutrient broth. Percentage inhibitions were calculated as:

$$\%\;\text{Biofilm}\;\text{inhibition}=\left[A\left(\text{control}\right)–A\left(\text{sample}\right)/A\left(\text{control}\right)\right]\times 100$$

#### 2.6.2 Biofilm formation inhibition assay

The glass slides were covered with nutrient broth and exposed to a clean bacterial strain culture overnight at 37°C. The slides were then washed with 2% crystal violet dye. Nutrient broth served as negative control whereas for positive control ciprofloxacin was employed. The capacity of the created extract/fractions to stop the growth of microbial biofilms was examined under a microscope.

### 2.7 Assessment of antidiabetic properties

#### 2.7.1 Antiglycation activity

Antiglycation activity was performed *in-vitro* to evaluate the phytochemicals nature present in *S. chirayita* nanosuspension and extract that can inhibit methyl glyoxal-induced fluorescence in BSA. The solution mixture of 45 mL of 67 mM sodium; phosphate buffer (pH 7.3), 450 mg of bovine serum albumin (BSA), and 4.5 milligrams of D-glucose was incubated for two days at 37°C. Afterward, measured the absorbance of a diluted 0.2 mL portion of the reaction mixture using a spectrophotometer with excitation and emission wavelength at 370 nm and 440 nm (BMS-UV-2600) respectively. Metformin was employed as a reference material, and for the negative control, the solution without D-glucose was used [13]. The test sample inhibition percentage was computed as:

$$\%\;\text{inhibition}=\left[A\left(440\text{nm}\right)/A\left(370\text{nm}\right)-A\left(440\text{nm}\right)\right]\times 100$$

#### 2.7.2 Alpha-amylase inhibition assay

In a 96-well plate, 30 μL of 20% (v/v) samples and standard acarbose were mixed together as a reference solution for evaluating the alpha-amylase inhibition activity. Then, amylase solution 10 μL was administered in each well with 0.02 M sodium phosphate buffer (pH = 7.0). 20 μL of 1M HCl and 75-microliter solution of iodine were then poured to each well. The wavelength of 630nm was used to measure the samples’ absorbance against the blank [23].

The below formula was used for % inhibition computation:

$$\%\;\text{inhibition}=A\left(\text{control}\right)–\text{A}\left(\text{sample}\right)/\text{A}\left(\text{control}\right)\times 100$$

### 2.8 Hemolytic assay (Preliminary cytotoxicity)

Initially, 3 mL of blood samples were subjected to centrifugation at 8000 rpm for 5 minutes for the separation of plasma. The resulting RBC pellets were then subjected to three rounds of centrifugation with 5 mL of cold PBS saline. Following that, the pellets were centrifuged at 8000 rpm for 5 minutes. In Eppendorf tubes, a diluted 180 μL of blood cell suspension was combined with 20 μL of each test sample and washed with PBS. PBS was used as a negative control, whereas 0.1% triton X-100 was applied as a positive control. Finally, an ELISA reader (BioTek, Winooski, VT, USA) was used to measure the absorbance at 570 nm [24]. The following formula was used to compute the % hemolytic inhibition:

$$\%\;\text{Hemolysis}=A\left(\text{Sample}\right)–A\left(\text{Negative}\;\text{Control}\right)/A\left(\text{Positive}\;\text{Control}\right)–A\left(\text{Negative}\;\text{Control}\right)\times 100$$

### 2.9 Statistical evaluation

One-way ANOVA (Analysis of variance) was applied to analyze the data and mean comparison of two populations i.e., extract and nanosuspension. The p-value of less than 0.05 indicated significance and results were stated as the percentage (%), mean, or standard deviation of the three replicated measurements.

## 3. Results

### 3.1 Structural characterization

#### 3.1.1 High-performance liquid chromatography

**Fig 1:**
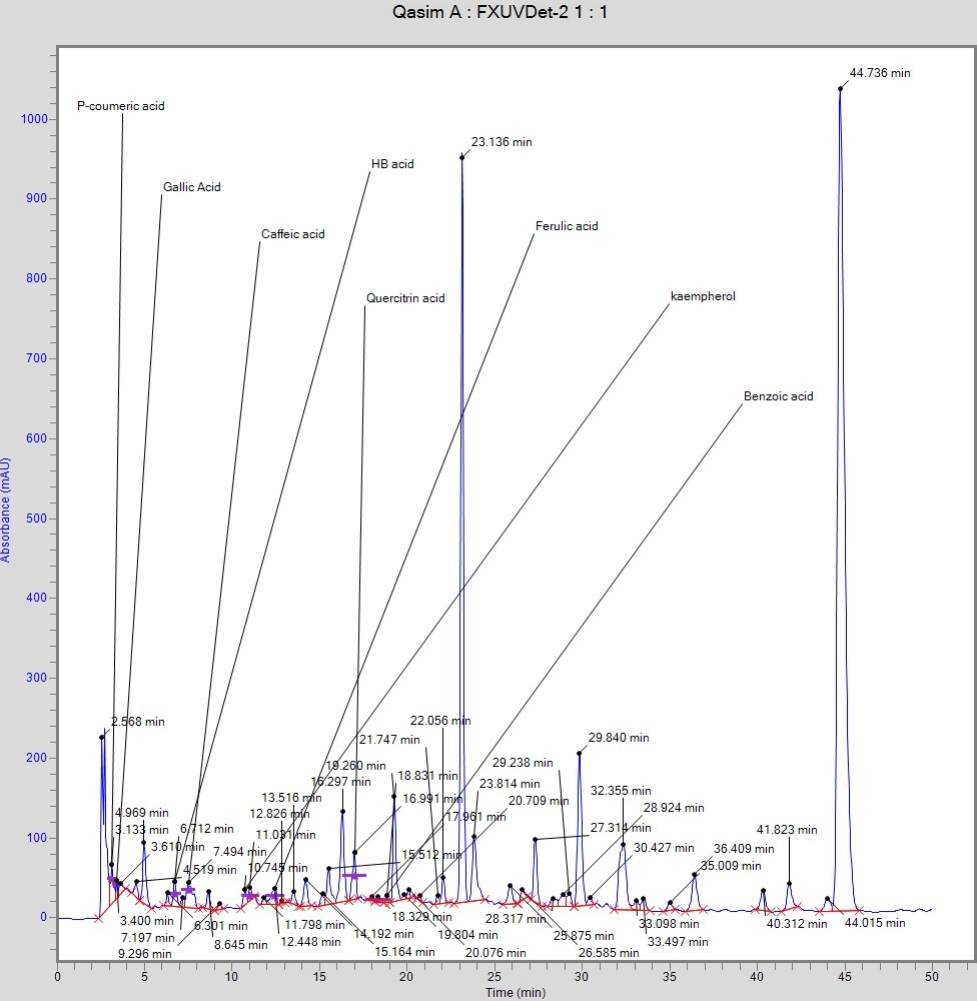
HPLC profile of *S. chirayita* whole plant extract.

The chromatogram of HPLC showed that only one flavonoid, quercetin, was detected in the sample. However, several phenolic compounds were identified, including p-coumaric acid, ferulic acid, gallic acid, and chlorogenic acid, based on the chromatogram analysis **(Fig 1)**.

**Table 1** shows the chromatograms generated by HPLC, illustrating the peaks of various active phytochemicals present in the *S. chirayita*. The following compounds, as evident in **Table 1** were detected in varying quantities: p-coumaric acid (64.37 ppm), gallic acid (1.5022 ppm), HB acid (4.987 ppm), caffeic acid (4.682 ppm), kaempferol (0.8695 ppm), ferulic acid (3.486 ppm), quercetin (57.2 ppm), and benzoic acid (0.3013 ppm). The values of these compounds are expressed in parts per million (ppm). Among these compounds, quercetin is a flavonoid, while p-coumaric acid and ferulic acid are phenolic compounds.

**Table 1:** Quantification of different flavonoids and phenolic compounds from *S. chirayita*.

Retention Time (min)	Compound Name	Area (mv. s)	Height	Amount (ppm)
3.133	P-coumeric acid	535700.5	48356.5	64.37
3.400	Gallic acid	171917.1	22527.3	1.5022
6.712	HB acid	430153.7	32180.1	4.987
7.494	Caffeic acid	799935.8	31040.1	4.682
10.745	Kaempherol	213141.2	18094.0	0.8695
12.448	Ferulic acid	454448.5	21089.9	3.486
16.991	Quercetin	821189.5	59797.1	57.2
18.831	Benzoic acid	87280.8	8664.7	0.3013

#### 3.1.2 Fourier-transform infrared spectroscopy

**Fig 2:**
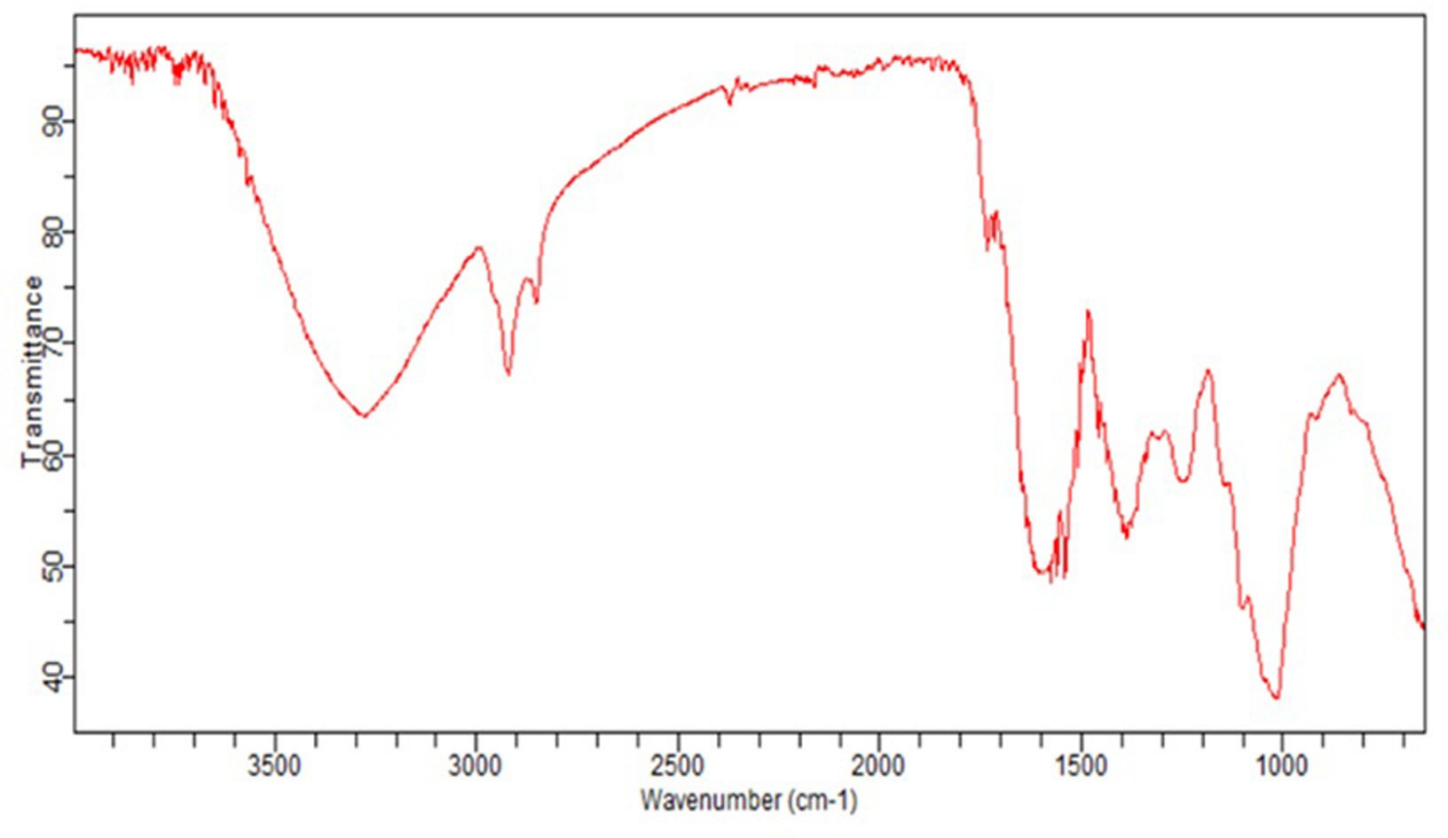
FTIR spectra of *Swertia chirayita* powder.

**Fig 2** and **Table 2** provide the predicted absorption values obtained from FTIR analysis, identifying various functional groups present in the powder of *S. chirayita*. The observed peaks and bands indicated that specific functional groups within the sample are present. The strong peak observed at 3278 cm-1 suggests the presence of alcohol in the sample. A medium peak observed at 2918 cm-1 indicates the existence of alkane functional groups. Another band at 2849 cm-1 show that both alkanes and aldehydes are present. Lastly, the medium bands observed at 1010 cm-1 suggest the presence of sulfoxide functional groups.

**Table 2:** FTIR spectrum chart indicating the identified functional groups in *S. chirayita* powder.

Peak no	Characteristic absorption	Identified Functional Groups	Compounds Class
1	3278	O-H stretching	Alcohols
2	2918	C-H stretching	Alkane
3	2849	C-H stretching	Alkane/Aldehyde
4	1010	S-O stretching	Sulfoxide

### 3.2 Antioxidant activity

**Table 3** shows the total phenolic content (TPCs) and total flavonoid content (TFCs) of *Swertia chirayita* extract and nanosuspensions. *S. chirayita* extract exhibited higher TPCs of 569.6 ± 7.8, whereas the nanosuspension demonstrated a slightly lower content of 500.6 ± 2.4. Regarding TFCs, the extract contained 368.5 ± 9.39, while the nanosuspension displayed a lower content of TFCs (229.5 ± 3.85). The assessment of DPPH radical scavenging activity revealed that *S. chirayita* extract exhibited high DPPH-free scavenging activity (28.9%) as compared to nanosuspensions (6%). These results suggest that the compounds present in the extract possess a longer-lasting bioavailability.

**Table 3:** Comparison of different assays of *S. chirayita* nanosuspension and extract.

Treatments	Antioxidant profile			Antidiabetic profile (%)		Biofilm inhibition (%)		Cytotoxicity (%)
	TPC	TFC	DPPH	Glycation inhibition	α-amylase inhibition	EC	SA	
SNS	500±7.8	229.5±3.85	06±3.61	35.8	5	69.12	40.33	33.63
SE	569.6±7.8	368.5±9.39	28.9±3.85	41.4	19.5	62.08	80.67	51.86
Control	750.87±6.63	244.44±2.63	89.56±0.00	56.91	82.53	59.39	42.01	96.45

* Results are represented as a percentage or as the mean and standard deviation of measurements taken in triplicate. SNS stands for *Swertia chirayita* nanosuspension, SE stands for *Swertia chirayita* extract, and *EC*: Escherichia *coli. SA*: *Staphylococcus aureus*. TPC: Total phenolic contents, TFC: Total flavonoid content, DPPH: 2,2-diphenyl l-picrylhydrazyl., ciprofloxacin (antimicrobial assay)., metformin (antiglycation assay)., and BHT (butylated hydroxytoluene)

### 3.3 Biofilm inhibitory potential

**Fig 3:**
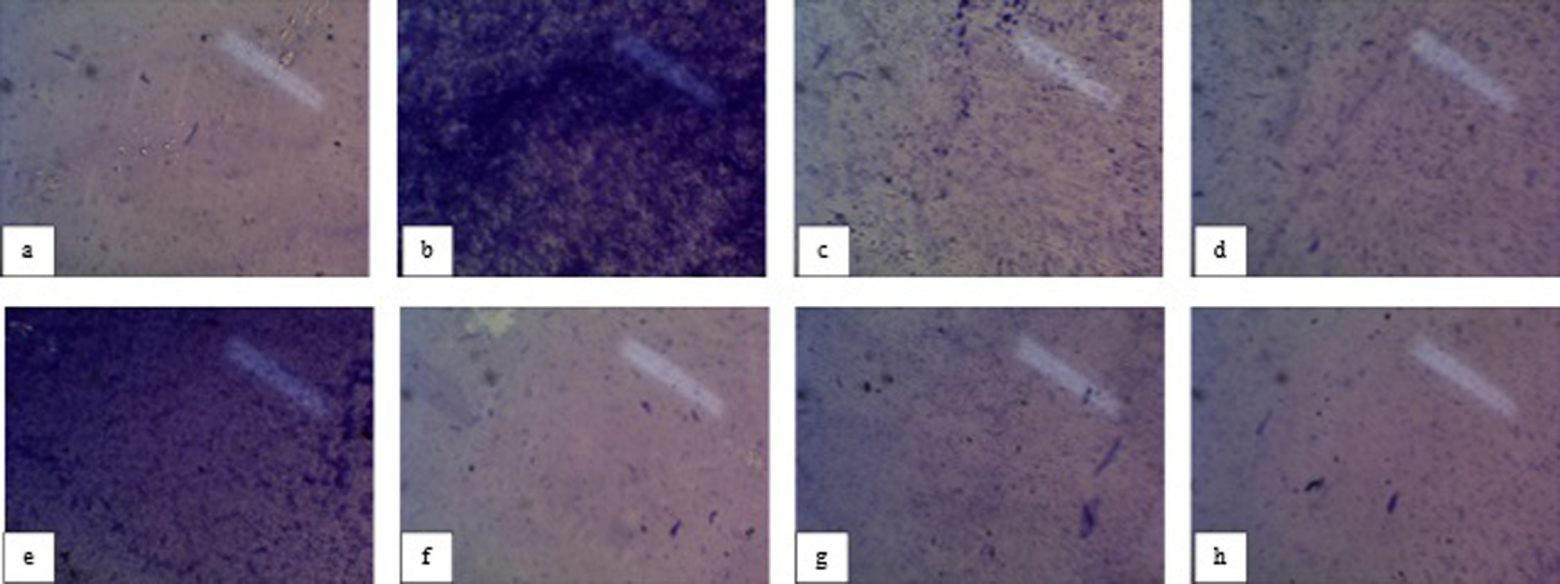
(a) Positive control *(E. Coli)* (b) Negative control *(E. Coli)* (c) (Qualitative assay) inhibition of *E. coli* by extract fraction (min.), (d) Inhibition of *E. coli* by nanosuspension fraction (max.), (e) Positive control *(S. aureus)* (f) Negative control *(S. aureus)* (g) Inhibition of *S. aureus* by extract fraction (min.), (h) Inhibition of *S. aureus* by nanosuspension fraction (max.).

Our current study found that *S. chirayita* extract exhibited a percentage inhibition of 62.08% against *E. coli*, while the nanosuspension demonstrated a higher percentage inhibition of 69.12% as given **(Fig 3)** and **Table 3**. In the case of *S. aureus*, the extract showed a percentage inhibition of 80.67%, whereas the nanosuspension displayed a lower percentage inhibition of 40.33%.

### 3.4 Antidiabetic potential

#### 3.4.1 Antiglycation potential

**Table 3** shows that ethanolic extract demonstrated a percentage inhibition of antiglycation 41.4%, while the nanosuspension displayed a slightly lower percentage inhibition of 35.8%.

#### 3.4.2 Alpha-amylase inhibition

**Table 3** shows that ethanolic extract demonstrated a higher percentage of alpha-amylase inhibition activity, 19.5% greater inhibition activity than the nanosuspension, which exhibited only a 5% inhibition activity.

### 3.5 Hemolytic potential

The results demonstrated that the plant extract of *S. chirayita* exhibited a significantly higher hemolytic activity of 51.86% compared to the nanosuspension, which showed a lower hemolytic activity of 33% **(Table 3)**.

## 4. Discussion

A study identified three major compounds from *S. chirayita* by HPLC, such as swertiamarin, mangiferin, and amarogentin. The retention times were approximately 8.5 min, 13.6 min, and 21.7 min, respectively [25]. In line with the findings of V Kumar’s study and previous research [26], also utilized HPLC analysis to investigate *S. chirayita*. According to their research, the plant contained gallic acid (0.562 ppm) and caffeic acid (0.568 ppm). Furthermore, a study corroborated the presence of various phytoconstituents in *S. chirayita* using HPLC analysis. The results of the study showed that the plant extract included quercetin, gallic acid, swertiamarin, ferulic acid, rutin, and amarogentin. The retention times for these compounds were determined to be 1.82 min, 2.29 min, 2.9 min, 15.57 min, 20.82 min, and 22.31 min, respectively [27]. Based on retention time and peaks, our investigation is in line with all these studies.

The FT-IR analysis of *Swertia chirayita* revealed several significant peaks corresponding to various functional groups. Notably, peaks at 3,610.74 cm-1, 3,089.96 cm-1, and 2,125.56 cm-1, indicated the presence of capping agents such as flavonoids, hydroxyl compounds, alcholic and phenolic compounds as well as primary and secondary amines. Additionally, a peak at 166.50 cm-1 and 864.11 cm-1 further supported the presence of these capping agents [28]. Expanding on this research, another group of scientists conducted an analysis of extracts of *Swertia chirayita* adsorbed on ZnONPs using FTIR to spot distinctive bands of functional groups. The analysis of ZnONPs derived from aqueous and alcoholic extracts revealed intriguing observations. Specifically, a peak at 2,931 cm−1 suggested the existance of C-H bonding arising from aromatic compounds. Furthermore, a band at 1,630 cm−1 indicated C = C bond stretching, typically associated with the alkenyl group. Notably, the peaks observed at 1,394 cm−1 and 1,378 cm−1 provided evidence of antioxidants, specifically polyphenols, as they represented C–F stretching vibrations [29]. In the present work, several functional groups in *S. chirayita* nanosuspensions were discovered, and the absorption predicted by the FTIR was validated.

Our findings agree with the earlier research. Previous research demonstrated that *S. chirayita* methanolic extract showed high phenolic contents (67.49 ± 0.5 mg GAE/g) [30]. Another experiment investigated the crude methanolic extract of *S. chirayita* and found that its different fractions exhibited high total phenolic contents and reported that total flavonoid contents in *S. chirayita* were higher than *Torilis leptophylla* [31].

The free radical scavenging activity of various plant extracts was calculated. Our findings are coherent with those of previous investigations. In a study, among different crude methanol extracts, *S. chirayita* showed significant DPPH free radical scavenging at a level of 95.56% at a concentration of 100 μg/ mL. Therefore, the IC50 value for *Swertia chirayita* was found to be lowest at 23.35 0.6 μg/mL. *S. chirayita* proved to be the best antioxidant among the different species [30].

The ethanol extract of *S. chirayita* contains active flavonoids and polyphenols. The highest antibacterial activity observed in the extract was consistent with the findings. Another investigation demonstrated the antibacterial activity of extracts. Significant activity was displayed by the methanol extract against B. subtilis (5 mm at 800 μg/mL) while only mild activity was exhibited by E. coli and S. typhi (4 mm at 800 μg/mL). It had less effect on P. mirabilis and B. polymyxa (1 mm at 800 g/mL), though [32].

According to previous study, both methanolic and aqueous leaf extracts of *S. chirayita* depicted strong % inhibition of alpha-amylase, i.e., 74.21±0.73* and 75.25±0.84* respectively [33]. A study investigated that ethanolic extract of *S. chirayita* exhibited 43% α-glucosidase inhibition. They also observed a significant difference between α-glucosidase inhibition of ethanolic and water solution (ρ<0.05) [34]. Scientists demonstrated the medicinal effect of *S. chirayita* in lowering glucose levels (14.5%) in diabetic individuals [35]. Similar to the above results, our study also confirmed that the *S. chirayita* extract had higher antiglycation activity than the nanosuspension.

A study revealed that a dosage of xanthone (1 and 5 M) derived from *S. chirayita* was helpful against cancer (EAC) and identified three major compounds from cells, MCF-7 and MDA-MB-231 cell lines without damaging the normal cells (fibroblast) [36]. Another investigation reported that ZnO-NPs synthesized from *S. chirayita* extract exhibit significant cytotoxic activity against cancer cells. On HCT-116, Caco-2, and HEK-293 cells, their IC50 values were found to be 34.356 ± 2.71 and 32.856 ± 2.99 μg/mL, 52.15 ± 8.23 and 63.1 ± 12.09 μg/mL, and 582.84 ± 5.26 and 615.35 ± 4.74 μg/mL, respectively [37].

## 5. Conclusion

This study focused to evaluate the medicinal efficacy of *Swertia chirayita* nanosuspension and extract and investigate the potential benefits of nanotechnology in enhancing the bioactivities of the whole plant nanosuspension. Biochemical characterization was carried out through spectroscopic analyses of HPLC and FTIR that confirmed the presence of bioactive compounds such as gallic acid, kaempherol and querctin. The results demonstrated extract contained higher TPCs (569.6 ± 7.8 mg GAE/100 g)) and TFCs (368.5 ± 9.39 mg CE/100 g) than *S. chirayita* nanosuspension TPCs (500.6 ± 7.8 500.6 ± 7.8 mg GAE/100 g)) and TFCs (229.5± 3.85 mg CE/100 g). Antioxidant activity was evaluated through DPPH scavenging assay, and nanosuspension exhibited a lower DPPH free radical scavenging potential (06 ±3.61) than extract (28.9± 3.85). Antiglycation and α-amylase inhibition assays were used to determine the antidiabetic activity of the nanosuspension and extract. Extract exhibited stronger (41.4%) antiglycation activity than 35.85% nanosuspension and 19.5% α-amylase inhibitory potential than 5% nanosuspension. In comparison to extract (62.08%), nanosuspension demonstrated greater biofilm inhibition efficiency against *E. coli* (69.12%). Extract showed high cytotoxicity potential (51.86%) than nanosuspension (33.63%). It was concluded that although the plant nanosuspension had a noticeable impact, the plant extract had superior antimicrobial, antioxidant, and antidiabetic properties and could be explored further for the development of new drugs.

## Supporting information

S1 File (PDF)

S1 Graphical abstract (JPG)

## References

[1] Khumalo GP, Van Wyk BE, Feng Y, Cock IE. A review of the traditional use of southern African medicinal plants for the treatment of inflammation and inflammatory pain. J Ethnopharmacol. 2022;283. doi: 10.1016/j.jep.2021.114436 34289396

[2] Ghosh S. Cisplatin: The first metal based anticancer drug. Bioorg Chem. 2019;88. doi: 10.1016/j.bioorg.2019.102925 31003078

[3] Luethi D, Liechti ME. Designer drugs: mechanism of action and adverse effects. Arch Toxicol. 2020;94: 1085–1133. doi: 10.1007/s00204-020-02693-7 32249347 PMC7225206

[4] Haq IU, Imran M, Nadeem M, Tufail T, Gondal TA, Mubarak MS. Piperine: A review of its biological effects. Phytotherapy Research. 2021;35: 680–700. doi: 10.1002/ptr.6855 32929825

[5] Alqahtani AS, Ullah R, Shahat AA. Bioactive Constituents and Toxicological Evaluation of Selected Antidiabetic Medicinal Plants of Saudi Arabia. Evidence-based Complementary and Alternative Medicine. 2022;2022. doi: 10.1155/2022/7123521 35082904 PMC8786507

[6] Cunningham AB, Brinckmann JA, Schippmann U, Pyakurel D. Production from both wild harvest and cultivation: The cross-border Swertia chirayita (Gentianaceae) trade. J Ethnopharmacol. 2018;225: 42–52. doi: 10.1016/j.jep.2018.06.033 29960022

[7] Mahendran G, Verma N, Singh S, Parveen S, Singh M, Luqman S, et al. Isolation and characterization of a novel xanthone from the hairy root cultures of Swertia chirayita (Roxb.) H. Karst. and its biological activity. Ind Crops Prod. 2022;176: 114369. doi: 10.1016/J.INDCROP.2021.114369

[8] Karak S, Nag G, De B. Metabolic profile and β-glucuronidase inhibitory property of three species of swertia. Revista Brasileira de Farmacognosia. 2017;27: 105–111. doi: 10.1016/J.BJP.2016.07.007/METRICS

[9] Zafar F, Jahan N, Asi MR, Ali S. Comparative evaluation of biological activities of native and nanosuspension of Terminalia arjuna Dietary Assessment of Patulin in fruits and juices View project The broad-spectrum antiviral recommendations for drug discovery against COVID-19 View project. Article in International Journal of Agriculture and Biology. 2019. doi: 10.17957/IJAB/15.0956

[10] Aslam S, Jahan N, Khalil-Ur-Rehman, Ali S. Formulation, optimisation and in-vitro, in-vivo evaluation of surfactant stabilised nanosuspension of Ginkgo biloba. J Microencapsul. 2019;36: 576–590. doi: 10.1080/02652048.2019.1662123

[11] Sahar P, Ali T, Naeem M, Hussain F. Nanotechnology approach for exploring the enhanced bioactivities, biochemical characterisation and phytochemistry of freshly prepared Mentha arvensis L. nanosuspensions. Phytochemical Analysis. 2022. doi: 10.1002/pca.3189 36453173

[12] Zafar F, Jahan N, Ali S, Jamil S, Hussain R, Aslam S. Enhancing pharmaceutical potential and oral bioavailability of *Allium cepa* nanosuspension in male albino rats using response surface methodology. Asian Pac J Trop Biomed. 2022;12: 26. doi: 10.4103/2221-1691.331792

[13] Ali T, Hussain F, Naeem M, Khan A, Al-Harrasi A. Nanotechnology Approach for Exploring the Enhanced Bioactivities and Biochemical Characterization of Freshly Prepared Nigella sativa L. Nanosuspensions and Their Phytochemical Profile. Front Bioeng Biotechnol. 2022;10: 685. doi: 10.3389/fbioe.2022.888177 35656198 PMC9152536

[14] Qiao Y, Wei Z, Qin T, Song R, Yu Z, Yuan Q, et al. Combined nanosuspensions from two natural active ingredients for cancer therapy with reduced side effects. Chinese Chemical Letters. 2021;32: 2877–2881. doi: 10.1016/J.CCLET.2021.03.049

[15] Liu Z, Smart JD, Pannala AS. Recent developments in formulation design for improving oral bioavailability of curcumin: A review. J Drug Deliv Sci Technol. 2020;60: 102082. doi: 10.1016/J.JDDST.2020.102082

[16] Dong Z, Wang R, Wang M, Meng Z, Wang X, Han M, et al. Preparation of Naringenin Nanosuspension and Its Antitussive and Expectorant Effects. Molecules 2022, Vol 27, Page 741. 2022;27: 741. doi: 10.3390/molecules27030741 35164006 PMC8837938

[17] Sahu T, Ratre YK, Chauhan S, Bhaskar LVKS, Nair MP, Verma HK. Nanotechnology based drug delivery system: Current strategies and emerging therapeutic potential for medical science. J Drug Deliv Sci Technol. 2021;63. doi: 10.1016/J.JDDST.2021.102487

[18] Mishra SB, Pandey H, Pandey AC. Nanosuspension of Phyllanthus amarus extract for improving oral bioavailability and prevention of paracetamol induced hepatotoxicity in Sprague–Dawley rats. Advances in Natural Sciences: Nanoscience and Nanotechnology. 2013;4: 035007. doi: 10.1088/2043-6262/4/3/035007

[19] Khezeli T, Daneshfar A, Sahraei R. A green ultrasonic-assisted liquid–liquid microextraction based on deep eutectic solvent for the HPLC-UV determination of ferulic, caffeic and cinnamic acid from olive, almond, sesame and cinnamon oil. Talanta. 2016;150: 577–585. doi: 10.1016/j.talanta.2015.12.077 26838445

[20] Manju S, Malaikozhundan B, Vijayakumar S, Shanthi S, Jaishabanu A, Ekambaram P, et al. Antibacterial, antibiofilm and cytotoxic effects of Nigella sativa essential oil coated gold nanoparticles. Microb Pathog. 2016;91: 129–135. doi: 10.1016/j.micpath.2015.11.021 26703114

[21] CHAHARDEHI AM, IBRAHIM D, SULAIMAN SF. Antioxidant Activity and Total Phenolic Content of Some Medicinal Plants in Urticaceae Family. Journal of Applied Biological Sciences. 2009;3: 27–31. Available: https://dergipark.org.tr/en/pub/jabs/issue/34904/387102

[22] Nawaz A, Ali T, Naeem M, Hussain F, Li Z, Nasir A. Biochemical, structural characterization and in-vitro evaluation of antioxidant, antibacterial, cytotoxic, and antidiabetic activities of nanosuspensions of Cinnamomum zeylanicum bark extract. Front Chem. 2023;11: 287. doi: 10.3389/FCHEM.2023.1194389 37214484 PMC10196027

[23] Unuofin JO, Otunola GA, Afolayan AJ. In vitro α-amylase, α-glucosidase, lipase inhibitory and cytotoxic activities of tuber extracts of Kedrostis africana (L.) Cogn. Heliyon. 2018;4: e00810. doi: 10.1016/J.HELIYON.2018.E00810 30294692 PMC6169336

[24] Powell WA, Catranis CM, Maynard CA. Design of self-processing antimicrobial peptides for plant protection. Lett Appl Microbiol. 2000;31: 163–168. doi: 10.1046/j.1365-2672.2000.00782.x 10972721

[25] Kumar V, Chauhan RS, Sood H. In Vitro Production and Efficient Quantification of Major Phytopharmaceuticals in an Endangered Medicinal Herb, Swertia chirata In Vitro Production and Efficient Quantification of Major Phytopharmaceuticals in an Endangered Medicinal Herb, Swertiachirata. International Journal of Biotechnology and Bioengineering Research. 2013;4: 495–506. Available: http://www.ripublication.com/ijbbr.htm

[26] Kshirsagar P, Chavan J, Nimbalkar M, Yadav S, Dixit G, Gaikwad N. Phytochemical composition, antioxidant activity and HPLC profiles of Swertia species from Western Ghats. 2014;29: 780–784. doi: 10.1080/14786419.2014.986124 25482162

[27] Lad H, Bhatnagar D. Amelioration of oxidative and inflammatory changes by Swertia chirayita leaves in experimental arthritis. Inflammopharmacology. 2016;24: 363–375. doi: 10.1007/s10787-016-0290-3 27738917

[28] Kumar M, Sinha MP. Green Nanotechnology: Synthesis of Silver Nanoparticles Using Aqueous Leaf Extract of Swertia Chirayita. 2017. Available: https://papers.ssrn.com/abstract=3955128

[29] Akhter SMH, Mahmood Z, Ahmad S, Mohammad F. Plant-Mediated Green Synthesis of Zinc Oxide Nanoparticles Using Swertia chirayita Leaf Extract, Characterization and Its Antibacterial Efficacy Against Some Common Pathogenic Bacteria. Bionanoscience. 2018;8: 811–817. doi: 10.1007/S12668-018-0549-9/FIGURES/9

[30] Khanal S, Shakya N, Thapa K, Pant DR. Phytochemical investigation of crude methanol extracts of different species of Swertia from Nepal. BMC Res Notes. 2015;8: 1–9. doi: 10.1186/S13104-015-1753-0/FIGURES/8 26708007 PMC4691535

[31] Atif Khan M, Zia M, Arfan M, Fatima N, Nazir A, Naseer M, et al. Antioxidants, antimicrobial and cytotoxic potential of Swertia chirata Synthesis of ter-butyl esters View project Simultaneous Hydrogen Production and Organic Pollutant Degradation View project Antioxidants, antimicrobial and cytotoxic potential of Swertia chirata. 2018. doi: 10.4066/biomedicalresearch.29-18-685

[32] Laxmi A, Siddhartha S, Archana M. ANTIMICROBIAL SCREENING OF METHANOL AND AQUEOUS EXTRACTS OF SWERTIA CHIRATA. Article in International Journal of Pharmacy and Pharmaceutical Sciences. 2011. Available: https://www.researchgate.net/publication/279850099

[33] Roy P, Abdulsalam FI, Pandey DK, Bhattacharjee A, Eruvaram NR, Malik T. Evaluation of Antioxidant, Antibacterial, and Antidiabetic Potential of Two Traditional Medicinal Plants of India: Swertia cordata and Swertia chirayita. Pharmacognosy Res. 2015;7: S57–S62. doi: 10.4103/0974-8490.157997 26109789 PMC4466770

[34] Phoboo S, Da Silva Pinto M, Barbosa ACL, Sarkar D, Bhowmik PC, Jha PK, et al. Phenolic-Linked Biochemical Rationale for the Anti-Diabetic Properties of Swertia chirayita (Roxb. ex Flem.) Karst. Phytotherapy Research. 2013;27: 227–235. doi: 10.1002/ptr.4714 22523004

[35] Ali S, Farooq M, Ali Panhwar W, Shoaib Ali C. Evaluation of hypoglycemic and hypolipidemic properties of Swertia chirata. entomoljournal.com. 2017;5: 1448–1451. Available: https://www.entomoljournal.com/archives/2017/vol5issue2/PartS/5-2-80-718.pdf

[36] Barua A, Choudhury P, Mandal S, Panda C, Saha P. Therapeutic potential of xanthones from Swertia chirata in breast cancer cells. Indian J Med Res. 2020;152: 285. doi: 10.4103/ijmr.IJMR_1153_18 33107489 PMC7881813

[37] Berehu HM, Anupriya S, Khan MI, Chakraborty R, Lavudi K, Penchalaneni J, et al. Cytotoxic Potential of Biogenic Zinc Oxide Nanoparticles Synthesized From Swertia chirayita Leaf Extract on Colorectal Cancer Cells. Front Bioeng Biotechnol. 2021;9: 1276. doi: 10.3389/fbioe.2021.788527 34976976 PMC8714927

