# Supplementary material for: Biochemical characterization of bioinspired nanosuspensions from Swertia chirayita extract and their therapeutic effects through nanotechnology approach
Source: PLoS One. 2024 Feb 8;19(2):e0293116. doi: 10.1371/journal.pone.0293116 (PMC10852254; doi:10.1371/journal.pone.0293116)
Supplement: S1 File — (PDF) [file pone.0293116.s001.pdf]

Sample Report - Single Channel

Sample Name	Qasim A		
Batch Group/Name	Hi-Tech/Qasim A		
Acquisition Method	Qasim A		
Processing Method	Qasim A		
Instrument Name	Instrument1	Channel Name	FXUVDet-2 1
Vial Number	1	Injection Number	1
Operator	Hi-Tech	Chromera Version	4.1.2.6410
Acquisition Date/Time	9/8/2021 2:05:49 PM		

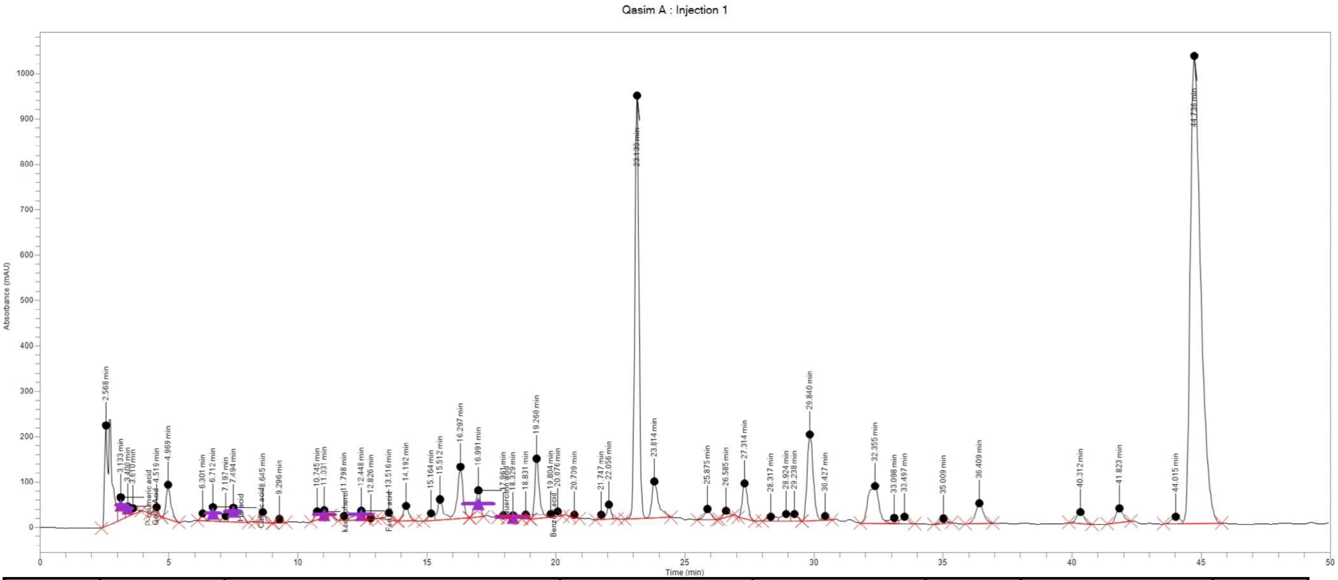

Peak #	RT (min)	Component Name	Area	Height	BL	Final Amount	Units
1	2.568		3,909,686.2	215,113.7	BV		
2	3.133	P-coumeric acid	535,700.5	48,356.5	VV		
3	3.400	Gallic Acid	171,917.1	22,527.3	VV		
4	3.610		210,310.7	14,245.3	VB		
5	4.519		222,177.3	19,653.1	BB		
6	4.969		970,666.4	75,503.0	BB		
7	6.301		169,411.6	16,473.5	BV		
8	6.712	HB acid	430,153.7	32,180.1	VV		
9	7.197		169,200.0	12,696.7	VV		
10	7.494	Caffeic acid	799,935.8	31,040.1	VB		
11	8.645		278,702.1	22,158.6	BB		
12	9.296		97,697.8	7,797.7	BB		
13	10.745	kaempherol	213,141.2	18,094.0	BV		

Sample Report - Single Channel

Peak #	RT (min)	Component Name	Area	Height	BL	Final Amount	Units
14	11.031		179,144.1	15,838.6	VB		
15	11.798		111,324.5	8,907.8	BV		
16	12.448	Ferulic acid	454,448.5	21,089.9	VB		
17	12.826		33,177.9	3,375.5	BB		
18	13.516		221,408.6	16,167.7	BB		
19	14.192		496,703.3	33,765.4	BB		
20	15.164		220,217.1	15,176.8	BV		
21	15.512		710,465.4	45,882.0	VV		
22	16.297		1,699,424.9	113,485.2	VB		
23	16.991	Quercitrin acid	821,189.5	59,797.1	BB		
24	17.961		58,477.4	5,652.4	BB		
25	18.329		94,974.8	6,467.7	BB		
26	18.831	Benzoic acid	87,280.8	8,664.7	BB		
27	19.260		1,626,643.7	133,020.8	BE		
28	19.804		61,467.0	6,039.8	EV		
29	20.076		113,080.3	10,363.6	VB		
30	20.709		74,513.5	6,827.1	BB		
31	21.747		121,468.0	11,428.7	BV		
32	22.056		371,326.1	33,120.7	VB		
33	23.136		10,571,334.3	929,375.8	BV		
34	23.814		1,448,820.6	81,286.0	VB		
35	25.875		405,015.5	23,640.0	BB		
36	26.585		194,102.3	13,605.0	BB		
37	27.314		1,089,935.2	79,037.8	BB		
38	28.317		253,742.9	10,170.5	BV		
39	28.924		325,346.9	14,614.2	VV		
40	29.238		267,259.3	15,826.9	VB		
41	29.840		3,125,414.7	192,151.9	BV		
42	30.427		139,206.2	9,449.2	VB		
43	32.355		2,319,858.8	82,480.9	BE		
44	33.098		243,059.8	12,267.1	EV		
45	33.497		293,354.5	15,593.4	VB		

Sample Report - Single Channel

Peak #	RT (min)	Component Name	Area	Height	BL	Final Amount	Units
46	35.009		172,482.9	9,820.4	BB		
47	36.409		939,204.6	45,286.3	BB		
48	40.312		502,390.2	25,385.3	BB		
49	41.823		604,469.7	31,420.0	BB		
50	44.015		380,500.5	16,371.7	BV		
51	44.736		29,097,843.1	1,030,778.0	VB		
Total			68,108,778.2				
