# Supplementary figures and images for: Biochemical characterization of bioinspired nanosuspensions from Swertia chirayita extract and their therapeutic effects through nanotechnology approach
Source: PLoS One. 2024 Feb 8;19(2):e0293116. doi: 10.1371/journal.pone.0293116 (PMC10852254; doi:10.1371/journal.pone.0293116)

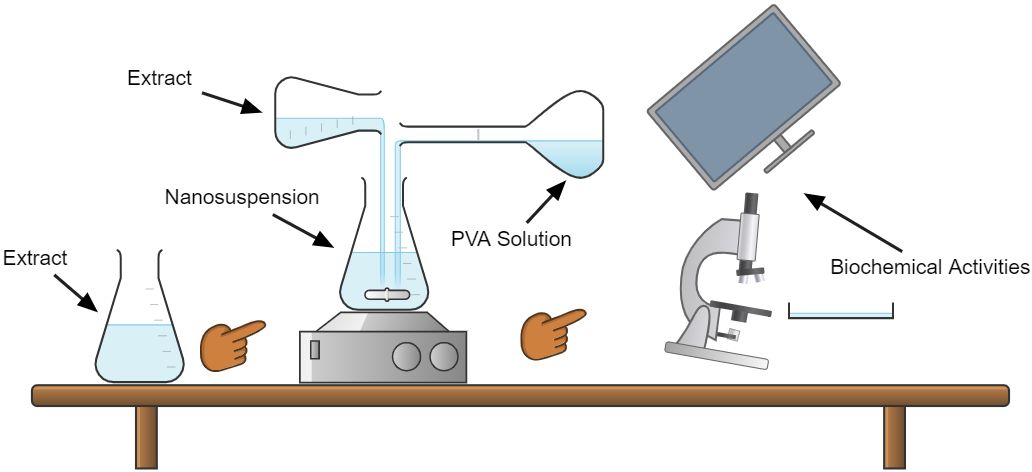

Supplement: S1 Graphical abstract — (JPG) [file pone.0293116.s002.jpg]
